# Description of a Large Family with Periodic Fever Carrying a Variant in *RXFP1* Gene: A Possible Novel Modulator of Inflammation in Autoinflammatory Diseases

**DOI:** 10.3390/ijms27020638

**Published:** 2026-01-08

**Authors:** Marianna Buttarelli, Giulia Rapari, Melania Riccio, Raffaele Manna, Donato Rigante, Eugenio Sangiorgi

**Affiliations:** 1 Sezione di Medicina Genomica, Department of Life Sciences and Public Health, Fondazione Policlinico Universitario Agostino Gemelli IRCCS, 00168 Rome, Italy; marianna.buttarelli@uniroma2.it (M.B.); giulia.rapari01@icatt.it (G.R.); melania.riccio01@icatt.it (M.R.); 2 Periodic Fevers Research Center, Università Cattolica Sacro Cuore, 00168 Rome, Italy; raffaele.manna@unicatt.it; 3 Sezione di Pediatria, Department of Life Sciences and Public Health, Fondazione Policlinico Universitario Agostino Gemelli IRCCS, 00168 Rome, Italy; donato.rigante@unicatt.it

**Keywords:** autoinflammatory diseases, PFAPA syndrome whole exome sequencing, personalized medicine

## Abstract

Autoinflammatory diseases involve recurrent systemic inflammation caused by dysregulated innate immunity, arising from genetic or multifactorial mechanisms, as seen in periodic fever, aphthous stomatitis, pharyngitis, and adenitis (PFAPA) syndrome. About 10% of PFAPA patients show autosomal dominant inheritance. We describe a three-generation family with a PFAPA-like recurrent fever syndrome displaying clear autosomal dominant transmission. All affected individuals tested negative on a diagnostic panel of 13 known autoinflammatory genes. Whole-exome sequencing was performed in two distantly related affected members, followed by variant filtering, segregation analysis, and phenotype-based prioritization. A single heterozygous missense variant in *RXFP1*, c.154G>A p.(Asp52Asn), co-segregated with disease in all affected relatives. This variant is extremely rare in population databases, absent from ClinVar, present in COSMIC, and predicted as damaging by REVEL and CADD. RXFP1, not previously implicated in autoinflammatory or innate immune disorders, encodes the relaxin family peptide receptor 1, a G protein–coupled receptor involved in extracellular matrix regulation, anti-fibrotic pathways, and modulation of inflammatory cytokine production. Protein network analysis showed interactions with RLXN1-3, inflammatory mediators, PTGDR, ADORA2B, and C1QTNF8, supporting an immunomodulatory function. This is the first report linking *RXFP1* variation to a hereditary recurrent fever syndrome, identifying relaxin signalling as a potential immune regulatory pathway.

## 1. Introduction

Autoinflammatory diseases (AID) are characterized by recurrent episodes of systemic inflammation without any identifiable infectious triggers and are generally attributed to dysregulation of the innate immune system; these disorders include recurrent fever syndromes, granulomatous conditions, and pyogenic diseases [1]. Differential diagnosis should be based on symptom duration and the pattern of specific organ involvement, such as serosal membranes, joints, skin, and gastrointestinal tract, often making diagnosis challenging [2].

Hereditary recurrent fever syndromes share intermittent episodes of fever lasting 3–21 days, occurring from several times a month to a few times per year; many affected individuals can develop AA amyloidosis over time if unrecognized or overlooked, mostly in the cases of familial Mediterranean fever, mevalonate kinase deficiency, tumour necrosis factor receptor-associated periodic syndrome (TRAPS), or cryopyrin-associated periodic syndrome [3,4]. Incomplete penetrance and overlapping features with other autoinflammatory or polygenic inflammatory disorders often make AID diagnosis challenging, as for the periodic fever, aphthous stomatitis, pharyngitis and adenitis (PFAPA) syndrome [5]. In particular, PFAPA syndrome was first described in 1987 as a childhood condition marked by clockwork-like periodic fever recurrence with aphthous ulcers, pharyngotonsillitis, and cervical lymphadenopathy [6]. Most of the patients present just with episodes of recurrent fever; they rarely present other signs or symptoms that could help to establish a definitive diagnosis. For these reasons, a new entity was coined in all those cases with just periodic fever defined as the syndrome of undifferentiated recurrent fever (SURF) [7]. Approximately 10% of PFAPA cases may show autosomal dominant inheritance, although the underlying genetic basis is not yet clarified [8]. In such situations, a broader genetic screening and eventually whole-exome sequencing (WES) may help to identify candidate genes. In a previous study, we analyzed four pedigrees with patients displaying a clinical diagnosis of PFAPA-like/SURF, with recurrent fever being the most notable manifestation, sequencing their exome and then assessing their segregating patterns; in each family, we identified potential new genes that could predispose to periodic fever in those families [9].

Herein, we report for the first time the co-segregation in a multigenerational family of a heterozygous variant in a new gene, *RXFP1*, never associated before with recurrent fevers, suggesting that *RXFP1* may play a previously unrecognized role in the pathophysiology of autoinflammatory diseases.

## 2. Case Report

**Figure 1:**
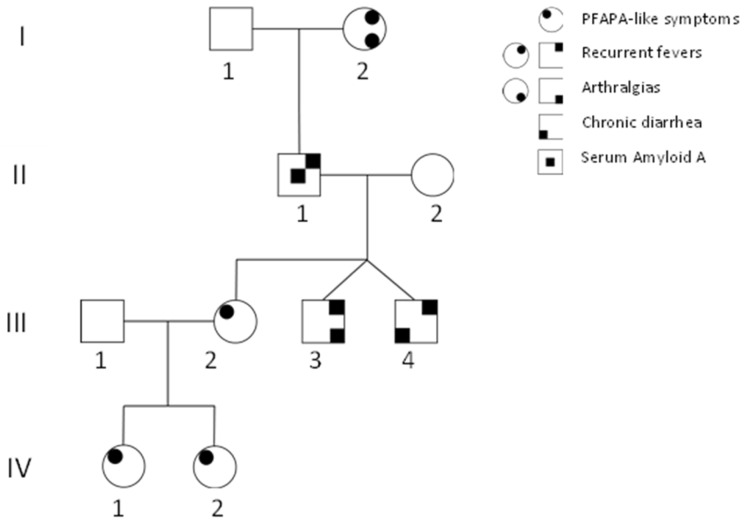
Pedigree of the family. The proband, IV2, came to our attention with a clinical diagnosis of PFAPA, as did her sister and her mother. Other family members also experienced recurrent fevers: in individuals I2 and II1, the fevers resolved around age 20, although arthralgias and persistently elevated serum amyloid A levels remained. Individual III4 had chronic diarrhea during childhood and now experiences only recurrent fevers. Roman numbers indicate different generations, Arabic numbers individuals in each generation.

All family members were recruited at the outpatient clinic of Periodic Fevers Research Centre in the Fondazione Policlinico Universitario A. Gemelli IRCCS. Clinical histories, biochemical profiles, and blood parameters were recorded during visits, including during febrile episodes and disease-free intervals. All participants provided a written informed consent, and the study was approved by the institutional ethics committee in 2020 (approval number ID3343, 25 June 2020). The proband (IV2), a two-year-old girl, presented with a PFAPA-like autoinflammatory phenotype. She had an older sister, a four-year-old with a similar presentation, as well as an extensive maternal family history of recurrent fever, affecting her mother, both maternal uncles (dizygotic twins), her maternal grandfather, and her great-grandmother (Figure 1).

**Figure 2:**
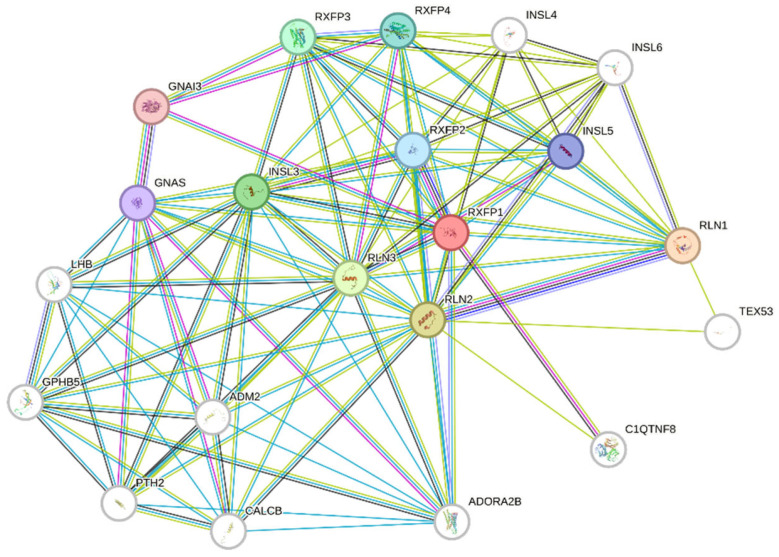
Cluster analysis of the RXFP1 protein in the STRING database. RXFP1 interacts with several ligands and other receptors; among them, C1QTNF8 and ADORA2B are involved in immune system regulation. Each circle represents a node, and the strength of each connection between nodes is indicated by the number of interconnecting lines. Different color connections indicate different level of interactions (see STRING for detailed description of each interaction); colored nodes indicate query proteins and first shell of interactors, white nodes second shell of interactors.

In several of these individuals, fever episodes were accompanied by additional signs and symptoms. Both sisters exhibited clockwork-like episodes of fever every four weeks, lasting 1–3 days, associated with adenitis, oral aphthosis, and malaise. For both girls, we proposed a clinical diagnosis of PFAPA. Their mother continued to experience recurrent febrile episodes. One uncle had joint pain in addition to fever, while the other had chronic diarrhea, with negative testing for common intestinal pathogens. Their grandfather no longer experienced fever but consistently showed elevated serum amyloid-A levels and progressive decline in renal function, as a probable consequence of amyloid deposits. His mother, now a 94-year-old, had not had a fever since early adulthood, but continued to report diffuse joint pain. For the remaining affected family members, a diagnosis of SURF was more appropriate. The proband tested negative for pathogenic variants on the 13-gene autoinflammatory disease panel. A *TNFRSF1A* variant (p.R92Q) was identified in all family members, both affected and unaffected, in homozygous and heterozygous states, supporting its benign role rather than a causative function. Additionally, it is classified as a variant of uncertain significance, and its high allele frequency in gnomAD (1.8% in the non-Finnish European population) strongly suggests it does not contribute to the phenotype observed in this family. Given the homogeneous phenotype across all family members and apparent autosomal dominant inheritance, whole-exome sequencing was performed for the individuals I2 and IV2. Whole-exome sequencing was performed by Dante Labs (L’Aquila, Italy). Sequencing achieved an average depth of ≥100×. FASTQ data were analyzed using standard workflows on the Galaxy platform. We retained heterozygous, protein-altering variants in coding or splice regions, passing filter quality, with minor allele frequency < 0.001 (gnomAD v4.0). We included in the final analysis all non-synonymous variants in exonic or splice-region sequences with a minor allele frequency < 0.001 in gnomAD. In total, 398 variants across 339 genes were identified in I4, and 129 variants across 94 genes in IV2. Final analysis selected exclusively the variants present in both women, suggesting their segregation through the family. From this analysis, only 56 variants in 40 genes were in common. To prioritize these genes separately, we used the VarElect web tool (http://ve.genecards.org, accessed on 1 November 2025), providing as input the gene list along with keywords related to autoinflammatory conditions: fever, autoinflammation, autoinflammatory disease, pyrin, inflammasome, innate immunity, and NLRP3. In addition, each variant was evaluated using gnomAD (https://gnomad.broadinstitute.org/, accessed on 1 November 2025). From this analysis, seven variants emerge (Table 1), and they are described with their in silico analyses (REVEL and CADD score). Both individuals were heterozygous for all variants. Segregation analysis was performed in the remaining affected members, IV1 and in both uncles of the three-generation family. Given the lack of segregation of the other variants among all the affected family members, the *RXFP1* variant (c.154G>A p.Asp52Asn) was considered the best candidate. This variant has not yet been reported in ClinVar. The same variant has been identified three times in the COSMIC database in two samples of colon cancer and in one skin sample of Merkel carcinoma. In all three samples, it has been confirmed to have a somatic origin, suggesting a potential functional role in dysregulation of the function of the RXFP1 receptor. Furthermore, REVEL and CADD scores classified the variant as potentially damaging to the protein function. The *RXFP1* gene has not previously been associated with inflammatory diseases; therefore, to explore any possible involvement in innate immunity pathways, we interrogated the STRING database (https://string-db.org/ accessed on 1 November 2025). Protein interaction network analysis through STRING indicated that RXFP1 interacts with related relaxin family receptors and ligands, RLN1 and 3, as well as with C1QTNF8, which has been implicated in regulating cellular motility in glioblastoma. RXFP1 also interacts with PTGDR and ADORA2B, both associated with immune modulation. In addition, relaxin signalling intersects with ADORA2B-mediated anti-inflammatory cytokine pathways (Figure 2).

## 3. Discussion

In this study, we describe a PFAPA-like autoinflammatory phenotype segregating in an autosomal dominant pattern across three generations, and we identify a previously unreported heterozygous missense variant in RXFP1 that co-segregates with disease. To our knowledge, this is the first evidence implicating the relaxin receptor pathway in a clinical scenario characterized by recurrent fevers. RXFP1, also known as LGR7, is a G-coupled extracellular receptor that was studied for its interaction with ligand RLN1 to RLN3 in the context of reproductive physiology, vasodilation, and extracellular matrix remodelling, particularly in fibrotic diseases [10]. However, accumulating evidence indicates that relaxin signalling also modulates systemic inflammation [11].

RXFP1 is the primary receptor mediating the pleiotropic actions of RLN2, which include significant anti-inflammatory and immunomodulatory effects on circulating blood cells [12]. Activation of RXFP1 in key inflammatory cells, such as mast cells and neutrophils, suppresses degranulation, reduces the production of superoxide anions, and limits the release of pro-inflammatory mediators, including interleukin IL6 and TNFα, often by engaging the nitric oxide cGMP signalling pathway [13,14]. Furthermore, RLN2 signalling through RXFP1 promotes the polarization of macrophages toward the anti-inflammatory tissue-repairing M2-like phenotype by decreasing the expression of pro-inflammatory M1-like markers and inhibiting the TLR4 nuclear factor NF-κB pathway [15]. Recombinant human relaxin mediates its anti-fibrotic actions, in part, by disrupting the pro-fibrotic interaction between transforming growth factor TGFβ1 and the NLRP3 inflammasome within myofibroblasts, which produces the pro-inflammatory cytokines IL1-β and IL-18 [16]. RXFP1 is essential for this suppression, operating through an RXFP1-nNOS-TLR-4-NLRP3 inflammasome-dependent mechanism in human cardiac myofibroblasts to inhibit the activity of the inflammasome [16]. Crucially, the RLX-induced suppression of NLRP3 inflammasome components, including NLRP3, pro-caspase-1, pro-IL-1β, and pro-IL-18, is annulled by the pharmacological blockade of RXFP1, confirming the receptor’s direct involvement in mediating this anti-inflammatory pathway [16].

The C1q-TNF-related protein 8 (CTRP8), characterized by its C1q-like domain, is identified as a ligand for the RXFP1 [17]. This CTRP8-RXFP1 signalling axis is specifically linked to pro-inflammatory events in the breast cancer tumour microenvironment, where CTRP8 activates RXFP1-expressing myeloid cells (such as macrophages) to promote M1-like polarization and stimulate the increased secretion of pro-inflammatory cytokines, such as IL-1β and TNFα [18]. RXFP1 interacts with other receptors such as ADORA2B and PTGDR within immune regulatory pathways, further supporting its role in immune homeostasis [19].

The RXFP1 gene encodes a G protein-coupled receptor with an N-terminal LDL receptor class A domain and then an extracellular leucine-rich repeat domain and a seven-transmembrane region; RXFP1 is unique among GPCRs, due to its N-terminal LDL-A module [20]. The length of the linker region between LDL-A and the LRR domain is critical for receptor activation [21]. An aberrant expression of RXFP1 has been associated with fibrotic disorders affecting the lung and skin [22].

The variant identified herein, p.Asp52Asn, affects a critical aspartic acid residue that serves as a binding site for Ca^2+^ ions; this residue is located in the N-terminal domain of the LDL receptor, which is essential for receptor activation [23]. The substitution may alter the receptor’s signalling threshold or bias downstream pathway engagement, potentially leading to episodic immune overactivation.

In the family studied, this variant was the only one that segregated among all seven affected individuals from our exome analysis. Moreover, the formal linkage score is just above two, insufficient for definitive linkage; co-segregation in seven individuals is highly suggestive. The pathogenic role of this variant is further supported by in silico predictions and by the fact, although this represents only circumstantial evidence, that it has been reported as a novel variant in three cancers: two colorectal cancers and one Merkel cell carcinoma [24].

## 4. Conclusions

Future directions should include functional assays of receptor signalling, ligand binding, and cytokine response, as well as exploration of RXFP1 modulation as a therapeutic target. Furthermore, the confirmation in additional families or sporadic cases would strengthen the case for RXFP1 as a novel autoinflammatory gene. Our findings suggest RXFP1 as a potential novel contributor to the development of an autoinflammatory disease. A clear limitation of this study is the lack of functional analysis. Further molecular and functional studies are warranted to determine the mechanistic role of relaxin signalling and to strengthen the evidence for the involvement of *RXFP1* in PFAPA syndrome and other autoinflammation-related disorders.

## Figures and Tables

**Table 1:** List of genes and variants segregating in IV2 and I2. For each variant, allele frequency (gnomAD) and predicted functional impact (CADD and REVEL scores) were evaluated. CADD scores above 20 and REVEL scores above 0.80 were considered significant. Only the variant in *RXFP1* segregated among all affected members of the family.

Gene	Variant	Allele Frequency in gnomAD	CADD	REVEL
*USP34*	p.Met2239Val	7/1609982	17.6	0.11
*RXFP1*	p.Asp52Asn	9/1614024	28.4	0.82
*GLIS3*	p.Asp840Asn	135/1614114	24.5	0.20
*SEC16B*	p.Gln500Ter	9/1607278	47.0	-
*LARP1B*	p.Asn644Ser	533/1610668	22.7	0.21
*ZC3H4*	p.Pro1189Arg	4/1609216	23.3	0.12
*VRK3*	p.Gly386Val	95/1613680	25.1	0.48

## Data Availability

The data presented in this study are available on request from the corresponding author.
